# The small GTPase ARF-1.2 is a regulator of unicellular tube formation in *Caenorhabditis elegans*

**DOI:** 10.1007/s12576-018-0617-5

**Published:** 2018-04-27

**Authors:** Eriko Kage-Nakadai, Simo Sun, Satoru Iwata, Sawako Yoshina, Yoshikazu Nishikawa, Shohei Mitani

**Affiliations:** 1 Department of Physiology, Tokyo Women’s Medical University School of Medicine, 8-1, Kawada-cho, Shinjuku-ku, Tokyo, 162-8666 Japan; 2 Graduate School of Human Life Science, Osaka City University, Osaka, 558-8585 Japan

**Keywords:** *Caenorhabditis elegans*, Unicellular tube formation, Small GTPase, ARF-1.2, CDC-42

## Abstract

The membrane trafficking events that regulate unicellular tube formation and maintenance are not well understood. Here, using an RNAi screen, we identified the small GTPase ARF1 homolog ARF-1.2 as a regulator of excretory tube formation in *Caenorhabditis elegans*. RNAi-mediated knockdown and knockout of the *arf*-*1.2* gene resulted in the formation of large intracellular vacuoles at the growth sites (varicosities) of the excretory canals. *arf*-*1.2* mutant animals were sensitive to hyperosmotic conditions. *arf*-*1.2* RNAi affected the localization of the anion transporter SULP-8, which is expressed in the basal plasma membrane of the excretory canals, but did not affect the expression of SULP-4, which is expressed in the apical membrane. The phenotype of *arf*-*1.2* mutants was suppressed by mutation of the small Rho GTPase CDC-42, a regulator of apical/basal traffic balance. These results suggest that ARF-1.2 plays an essential role in basal membrane traffic to regulate the formation of the unicellular excretory tube.

**Electronic supplementary material:**

The online version of this article (10.1007/s12576-018-0617-5) contains supplementary material, which is available to authorized users.

## Introduction

Epithelial tubes that enable nutrition uptake and fluid transport are essential in all metazoans. Tube formation requires cell polarization and the maintenance of two distinct domains, namely, the apical and basal membranes. The small GTPase family members play essential roles in the multiple steps required for polarized membrane traffic and multicellular tube formation. For example, the small GTPase RAB11, a member of the Rab family, has been implicated in the regulation of the apical recycling pathway [1]. In addition, a member of the small GTPase Arf/Sar family, ARF6, has been proposed to act in clathrin-dependent endocytosis at the apical [2] and basolateral [3] membranes of polarized epithelial cells such as Madin-Darby Canine Kidney (MDCK) cells. CDC42, a member of the Rho family of small GTPases, has been shown to function in a pathway that defines the apical membrane. Specifically, CDC42 has been shown to recruit the Par complex to the apical membrane [4] and is required for the multicellular apical polarization that drives and maintains multicellular tubes [5].

Contrary to multicellular tubes, unicellular tubes, including capillaries, are composed of individual cells with a hollow lumen. The *Caenorhabditis elegans* (*C. elegans*) excretory system provides a simple model of unicellular tube morphogenesis [6–8]. The excretory cell, a single cell that forms the major tubular component, extends branched processes along the length of the body to regulate fluid osmolarity and ion content. WNK kinases, CLIC-like proteins, Patched-related proteins, mucins, and aquaporins have all been reported to participate in the development and function of the excretory cell [9–11]. Transcription factors, such as CEH-6, NHR-31, and PROX-1, have also been demonstrated to control downstream genes to form the excretory cell [12–14].

Several genes that are related to membrane traffic have also been proposed to play roles in excretory tube formation. For example, mutants for *rdy*-*1*/*vha*-*5*, which encodes a vacuolar H^+^-ATPase α-subunit, display less extension of the excretory tubes [15]. In addition, loss-of-function mutations in *exc*-*5*, which encodes a homolog of FDG1 RhoGEF (guanine exchange factor), cause abnormalities in the apical membrane of the excretory cell [16]. However, the molecular mechanisms that regulate intracellular polarized transport in unicellular tubes are still largely unknown. In the present study, we identified the small GTPase ARF-1.2 as a regulator of basal trafficking in the excretory tube of *C. elegans*.

## Results and discussion

### The small GTPase ARF-1.2 is a regulator of excretory tube formation and function

**Fig. 1:**
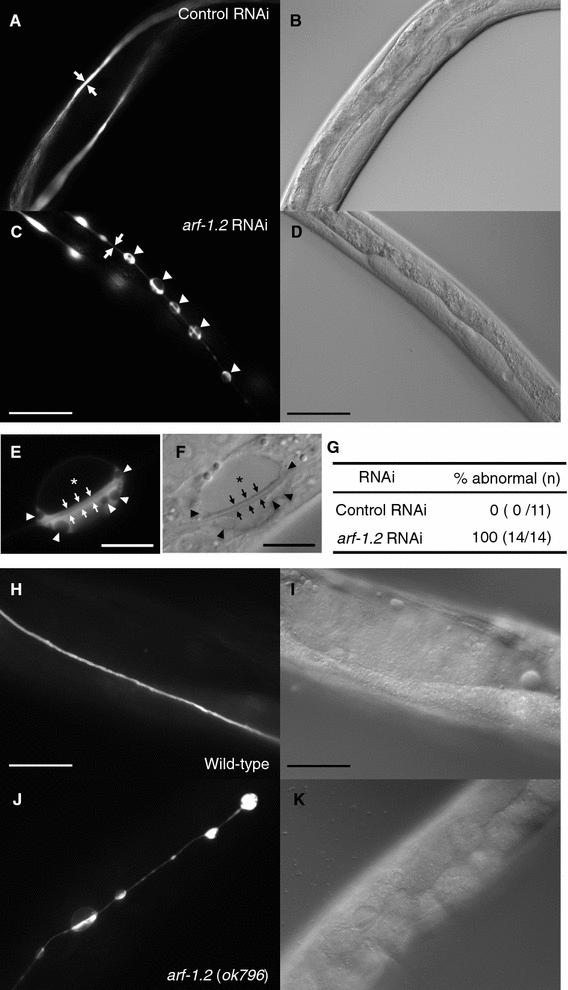
*arf*-*1.2* RNAi affects the morphology of the excretory cell. **a**–**d***tmIs807 [hmit*-*1.2p::GFP]* animals expressing GFP in the excretory cell (P0) were treated by control RNAi (**a**,** b**) or the *arf*-*1.2* RNAi (**c**, **d**). L2 hermaphrodites (F1) were photographed. Fluorescence (**a**, **c**) and DIC (**b**, **d**) images. The* arrows* indicate the canal tubes. The canals of the *arf*-*1.2*-treated animals were thinner (**c**) than those of the control animals (**a**). The* arrowheads* indicate vacuoles in the excretory canals (**c**). **e**, **f** Magnified fluorescence (**e**) and DIC (**f**) images of the vacuole observed in the *arf*-*1.2* RNAi. The* arrows* indicate the lumen. Besides a major vacuole (*asterisk*), small vacuoles (*arrowheads*) were observed in the photographed animal.* Scale bars* = 10 µm. (**g**) The percentages of animals that displayed abnormal morphology of the excretory canals. (**h**–**k**) *tmIs806 [hmit*-*1.2p::GFP]* animals expressing GFP in the excretory cell. Wild-type animals (**h**, **i**) or the *arf*-*1.2* mutants (**j**, **k**). Fluorescence (**h**, **j**) and DIC (**i**, **k**) images. Adult hermaphrodites were photographed.* Scale bars* = 50 µm

We previously reported that a H^+^/myo-inositol transporter (HMIT) gene, *hmit*-*1.2,* was selectively expressed in the excretory cell and the sheath glia in *C. elegans* [17]. To identify the gene(s) that participates in membrane traffic of the unicellular tubes, we carried out an RNAi screen for clone(s) that affect the morphology of the excretory cell. To this end, we used *hmit*-*1.2p::egfp* transgenic animals that express EGFP in the excretory cell, and an RNAi clone sub-library for membrane traffic-related genes (listed in Table S1) that were prepared by the Ahringer Library. We found that RNAi targeting the *C. elegans ARF1* homolog *arf*-*1.2* (*B0336.2*) caused vacuoles and thin excretory canal tubes (100%, *n* = 14) compared with control RNAi (0%, *n* = 11) (Fig. 1a–d). Magnified images revealed that the luminal structure remains and vacuoles exist intracellularly (Fig. 1e, f). Vacuoles with the major and shorter axis of 12.6 ± 5.2 and 7.5 ± 3.2 µm (± SD, *n* = 47), respectively, were observed along the canal at an average number of 2.9 per 100 µm. Although two *ARF1* homologs have been identified in worms, with varying levels of sequence identity to human ARF1 (ARF-1.1 61% identity; ARF-1.2, 94% identity) [18], RNAi against the *arf*-*1.1* (F45E4.1) gene did not obviously affect the morphology of the excretory canals (Table S1).

To determine whether the *arf*-*1.2* gene is responsible for this phenotype, we assayed *arf*-*1.2*(*ok796*) deletion mutants for the morphology of the excretory cell. Notably, *arf*-*1.2* mutants displayed a phenotype that is indistinguishable from that of *arf*-*1.2* RNAi-treated animals (Fig. 1). These results strongly suggest that *arf*-*1.2* is required for proper tube formation of the excretory canals. To further characterize the abnormal excretory cell of the *arf*-*1.2* mutants, transmission electron microscopy (TEM) was performed. Although vacuoles failed to be captured, we noticed that the *arf*-*1.2* mutant exhibited squashed excretory canals, in which the lumen and canaliculi were poorly defined (Fig. S1). One possibility is that the abnormal formation of the *arf*-*1.2* excretory canals may cause reduced fluid excretion to the lumen, resulting in the squashed lumen in the process of TEM, although other possible explanations cannot be ruled out.

**Fig. 2:**
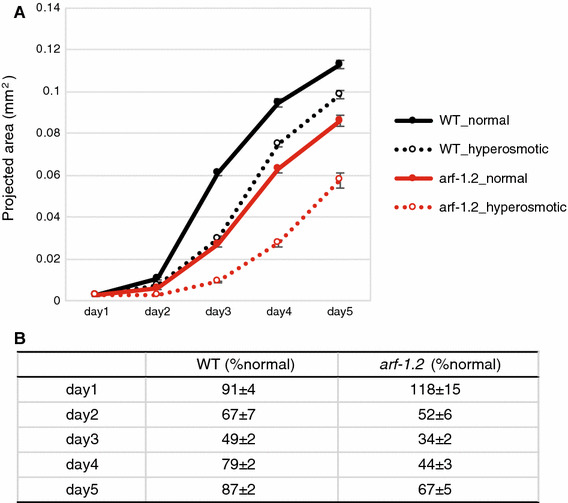
*arf*-*1.2* mutants were sensitive to hyperosmotic stress. **a** Body size of the wild-type (*black circles*) and *arf*-*1.2* mutant animals (*red circles*) was measured under the normal (*solid lines*) and hyperosmotic condition (*broken lines*). Each group contained more than 15 animals. The* error bars* indicate the SEM. **b** The percentages of body sizes under hyperosmotic conditions in comparison to those under normal conditions. ± SEM

Intracellular vacuoles detected following inactivation of the *arf*-*1.2* gene were regularly arrayed throughout the excretory canals. These vacuoles were reminiscent of the varicosities that are considered to be growth sites of the canals [13] and which are frequently observed during L1 larval stages and under hyperosmotic conditions [19]. The excretory system is composed of excretory, duct, and pore cells, which are essential for maintaining osmolarity homeostasis [20]. Together with TEM results, we speculated that *arf*-*1.2* may play a role in resistance to osmotic stress. To test this hypothesis, the growth (body size) under hyperosmotic conditions was measured. The body size of the *arf*-*1.2* mutant was highly reduced by hyperosmotic stress when compared with that of wild-type animals (Fig. 2). These results suggest that *arf*-*1.2* may be involved in the function of the excretory tube to maintain osmolarity homeostasis.

### Subcellular localization of ARF-1.2

**Fig. 3:**
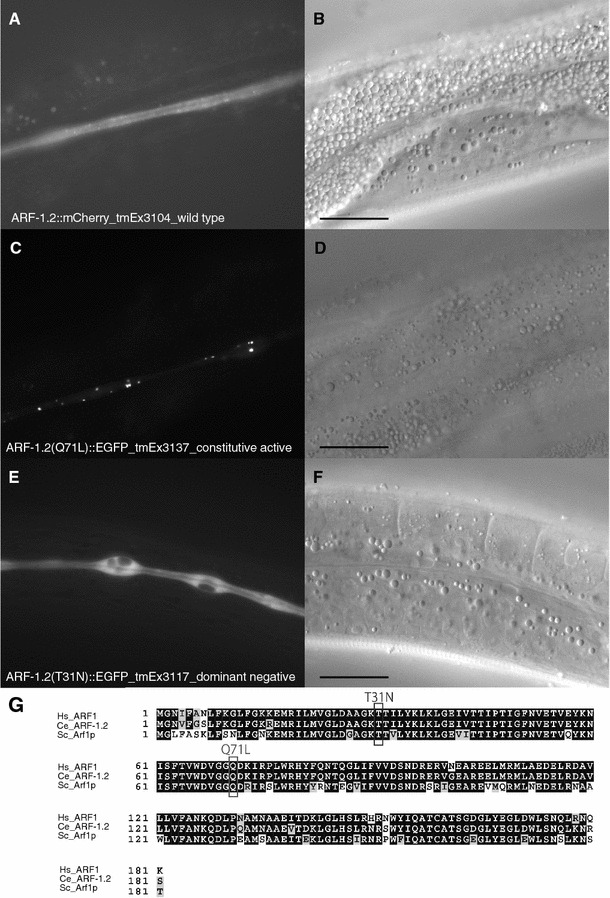
The subcellular localization of ARF-1.2 in the excretory canals. **a**, **b** The wild-type ARF-1.2 displayed cytosolic distribution with a partially punctate pattern. **c**, **d** The constitutively active form ARF-1.2(Q71L) exhibited a punctate pattern. No vacuoles were observed. **e**, **f** The dominant negative form ARF-1.2(T31N) showed cytosolic distribution in the excretory canals. The* arrowheads* indicate vacuoles. Young adult *tmEx3104* (for the wild type), *tmEx3137* (for QL form), and *tmEx3317* (for TN form) hermaphrodites were photographed.* Scale bars* = 20 µm. **g** Alignment of the *Homo sapiens* (Hs) ARF1, *C. elegans* (Ce) ARF-1.2 and *Saccharomyces cerevisiae* (Sc) Arf1p. The* numbers* indicate the positions of amino acid residues. Fully conserved amino acid residues are indicated by a reverse background, and similar but not identical residues are* shaded*

ARF1 localizes at the Golgi network and is a key regulator of vesicle trafficking from the trans-Golgi network to both the plasma membrane and the endosomes in yeast and mammals [21]. However, the subcellular localization of *C. elegans* ARF-1.2 is unknown. To determine the localization of ARF-1.2 in the excretory cell, ARF-1.2 fusion proteins were expressed under the *vha*-*8* promoter (Fig. 3). ARF-1.2(WT) cells exhibited cytosolic distribution and a partial punctate pattern (Fig. 3a, b). Meanwhile, cells expressing ARF-1.2(Q71L), a constitutively active form of ARF-1.2, showed a punctate pattern (Fig. 3c, d, g). To mark subcellular compartments in the excretory canals, we generated organelle marker strains, AMAN-2 (for Golgi bodies), RAB-5 (for early endosomes), RAB-7 (for late endosomes), RAB-11 and RME-1(for recycling endosomes), and LMP-1 (for lysosomes), as shown in Fig. S2. Among those markers, a Golgi marker AMAN-2 displayed punctate localization throughout the canals that resembled the pattern of constitutively active ARF-1.2(Q71L) (Fig. 3c, Fig. S2). The punctate pattern of AMAN-2 is compatible with that of GRIP, another Golgi marker [16]. ER/Golgi distribution throughout the excretory canals was confirmed by transmission electron microscopy and tomography [14]. Taken together, our data are compatible with the idea that ARF-1.2 localizes, at least in part, to the Golgi in the excretory canals. We also found that the overexpression of the dominant negative ARF-1.2(T31N) occasionally caused vacuole formation (Fig. 3e–g), supporting a model in which ARF-1.2 activity is required for proper tube formation of the excretory canals.

### *arf*-*1.2* RNAi affected the localization of the basal membrane protein

**Fig. 4:**
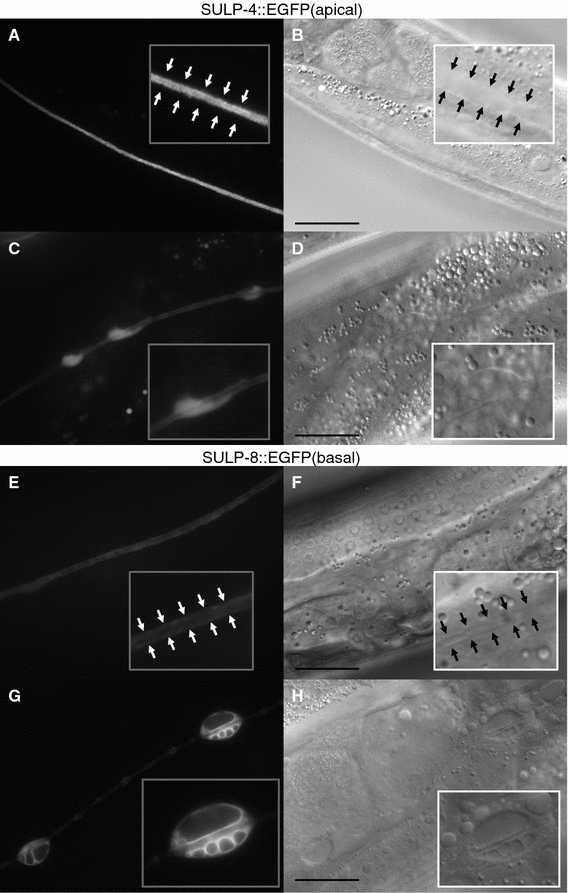
The localization of the basal membrane anion transporter SULP-8 was affected by *arf*-*1.2* RNAi. **a**–**d***tmEx3093 [sulp*-*4p::SULP*-*4::EGFP]* animals were treated with control RNAi (**a**, **b**) or the *arf*-*1.2* RNAi (**c**, **d**). **e**–**h***tmEx3096 [sulp*-*8p::SULP*-*8::EGFP]* animals were treated with control RNAi (**e**, **f**) or the *arf*-*1.2* RNAi (**g**, **h**). Fluorescence (**a**, **c**, **e**, **g**) and DIC (**b**, **d**, **f**, **h**) images. Magnified images are shown in the insets. The* arrowheads* indicate the basal membrane of the excretory canals.* Scale bars* = 20 µm. SULP-4::EGFP and SULP-8::EGFP localized at the apical membrane and basal membrane, respectively. Intracellular vacuoles with multi-compartments were labeled by SULP-8::EGFP but not by SULP-4::EGFP

We next sought to identify the origin of the intracellular vacuoles observed following inactivation of *arf*-*1.2*. To address this question, transgenic animals expressing subcellular markers were treated with *arf*-*1.2* RNAi (Fig. S2). We found that the membranes of the intracellular vacuoles were labeled by LMP-1::GFP (Fig. S2). Although LMP-1::GFP is localized to lysosomes in coelomocytes [22], it has been reported that LMP-1::GFP localizes to unidentified organelles that likely originate from endosomes [23] and that it primary labels basolateral membranes in other tissues, such as the intestines [24]. Therefore, we speculated that the intracellular vacuoles originated from the basal plasma membrane or from trafficking cargo to the basal membrane in the excretory canals. To test this possibility, the localization of the basal membrane anion transporter SULP-8 and the apical membrane protein SULP-4 [25] was examined. Notably, *arf*-*1.2* RNAi affected the localization of SULP-8, and SULP-8::EGFP labeled the membranes of vacuoles. On the other hand, the localization of SULP-4::EGFP was less affected (Fig. 4). These results suggest that ARF-1.2 plays a role in basal membrane traffic in the excretory canals.

### The vacuolar phenotype of *arf*-*1.2* mutants was suppressed by mutation of the small Rho GTPase CDC-42

ARF1 has been proposed to play a role in polarized transport through the dynamic control of AP1 and AP4 coat assembly in the trans-Golgi network (TGN) [26]. Because of the lack of an AP4 complex in *C. elegans*, AP1-clathrin components were knocked down and assayed for phenotypic abnormalities. However, we were not able to determine whether RNAi of the AP1-clathrin components (*chc*-*1*, *apg*-*1*, *aps*-*1*, *apb*-*1*) exhibited canal defects due to the severe Gro or Let phenotypes. ARF-1.2 and the ArfGEF GBF-1 have been proposed to act in ER-mitochondrial contacts to regulate mitochondrial morphology and function [27]. Skorobogata et al. has reported that ARF-1.2 and the ArfGEF AGEF-1 antagonize LET-23 EGFR basolateral membrane localization and signaling in the vulva [28]. As vacuoles in the excretory canals were not detected following RNAi against *gbf*-*1* or *agef*-*1*, other ArfGEF(s) may participate in the function of ARF-1.2 in the excretory canals. However, we were not able to rule out the possibility that RNAi against *gbf*-*1* and *agef*-*1* were not sufficiently effective.

**Fig. 5:**
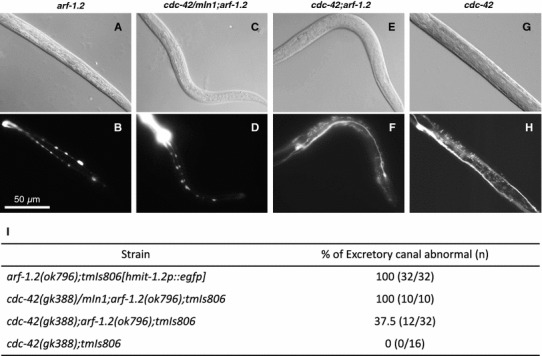
The vacuolar phenotype of *arf*-*1.2* mutants was suppressed by the *cdc*-*42* mutation. **a**–**h** L4 larva or young adult animals from *tmIs806[hmit*-*1.2p::egfp]* under the *arf*-*1.2(ok796)* genetic background (**a**, **b**), *cdc*-*42(gk388)/mIn1;arf*-*1.2(ok796)* (**c**, **d**), *cdc*-*42(gk388);arf*-*1.2(ok796)* (**e**,** f**), and *cdc*-*42(gk388)* animals (**g**, **h**) were photographed. Fluorescence (**a**, **c**, **e**, **g**) and DIC (**b**, **d**, **f**, **h**) images.* Scale bar* = 50 µm.** i** The percentages of animals that exhibit multiple vacuoles in the excretory canals

Next, to understand the molecular mechanisms underlying the *arf*-*1.2*-dependent regulation of excretory tube formation, suppressor gene(s) were surveyed. As a result, we found that mutation of *cdc*-*42*, which encodes the small Rho GTPase CDC42, suppressed the vacuolar phenotype of the *arf*-*1.2* mutants (Fig. 5). CDC42 plays multiple roles in vesicle transport in epithelial cells and can regulate apical/basolateral traffic [4]. In the *C. elegans* excretory canals, CDC-42 has been proposed to participate in apical transport with WSP-1, the G-protein-binding protein WASP homolog [16]. Taken together, our results suggest that the downregulation of apical transport by the inactivation of CDC-42 may restore basal transport in *arf*-*1.2* mutants.

### Model of membrane traffic in the excretory unicellular tube

**Fig. 6:**
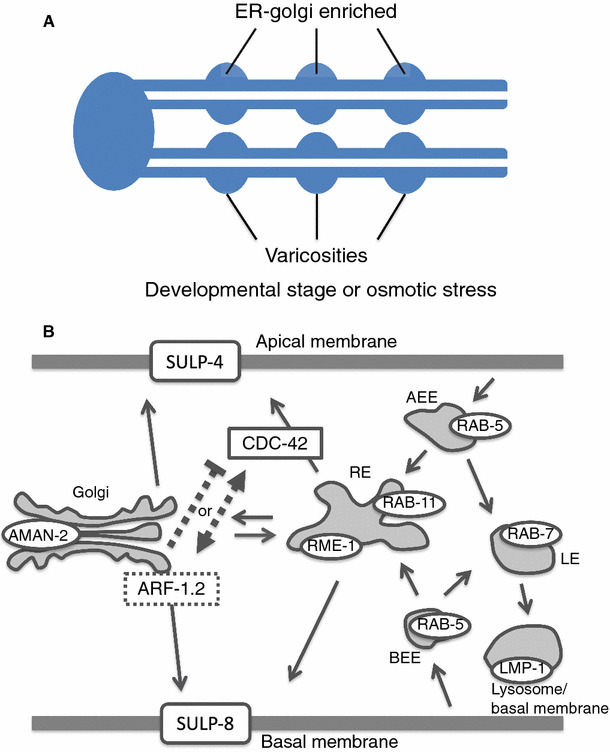
Working model for the role of ARF-1.2 in the polarized membrane traffic in the excretory canals. **a** Schematic structure of the excretory cell. Varicosities, which are frequently observed during L1 larval stages and under hyperosmotic conditions, contain ER-Golgi networks and are considered to be growth sites of the canals. **b** Model of polarized membrane traffic in the excretory canals.* Solid lines* show established trafficking pathway in the previous study [16] and* broken lines* indicate hypothetical mechanisms based on the present study. *RE* recycling endosome, *AEE* apical early endosome, *BEE* basal early endosome, *LE* late endosome

Varicosities, where ER-Golgi networks are enriched [14], are considered to be growth sites of the canals [13] and are frequently observed during L1 larval stages and under hyperosmotic conditions [19] (Fig. 6a). The fact that RNAi-mediated knockdown and knockout of the *arf*-*1.2* gene caused large vacuoles at the varicosities of the excretory canals seems to reflect the importance of ARF-1.2-regulated membrane traffic at the varicosities.

Mattingly and Buechner described a model of membrane traffic in the excretory canals, with organelle markers GRIP (for Golgi bodies), RAB-5 (for early endosomes), RAB-7 (for late endosomes), RAB-11 and RME-1(for recycling endosomes), and GLO-1 (for lysosomes), in which CDC-42 regulates the transport from the recycling endosome to apical plasma membrane [16]. In the present study, subcellular compartments were labeled with those markers, except for Golgi marker (AMAN-2 in this study) and lysosome marker (LMP-1 in this study), suggesting that ARF-1.2 localizes, at least in part, to the Golgi bodies in the excretory canals. We showed that the inactivation of *arf*-*1.2* caused accumulation of intracellular vacuoles that are likely to be related to basal membrane trafficking. In addition, the vacuolar phenotype of *arf*-*1.2* mutants was suppressed by mutation of the *cdc*-*42* gene. The result implicates an interplay between ARF-1.2 and CDC-42, in which ARF-1.2 suppresses or interact with CDC-42 directly or indirectly to balance of the apical and basal transport. Based on these data, we propose a working model, in which ARF-1.2 regulates basal membrane traffic of the excretory canals (Fig. 6b).

ARF1 has been reported to function in epidermal cell polarity in* Arabidopsis* [29]. However, the role of ARF1 in polarized transport has been less explored than that of ARF6. In the present study, we showed that the *C. elegans* ARF1 homolog ARF-1.2 plays a role in basal membrane traffic in the excretory unicellular tube and in the morphology of the canals. Our findings provide new insights into the function of ARF1 in polarized transport and unicellular tube formation.

## Experimental procedures

### Strains

*C. elegans* strains were cultured using standard techniques [30]. Bristol strain N2 was used as the wild-type *C. elegans* strain. The following strains were obtained from the Caenorhabditis Genetics Center: VC567 *arf*-*1.2(ok796) III*, VC898 *cdc*-*42(gk388)/mIn1 [mIs14 dpy*-*10(e128)] II.*

### Constructs and transgenic lines

*tmIs806[hmit*-*1.2p::egfp] and tmIs807[hmit*-*1.2p::egfp]* was generated in the previous report [31]. To generate the *vha*-*8p::EGFP* plasmid, the *vha*-*8* upstream genomic fragment (approximately 1.5 kb) was PCR amplified and cloned into the BamHI/NotI sites of the pFX_EGFPT expression vector [32]. To generate the *vha*-*8p::ARF*-*1.2::EGFP* plasmid, *arf*-*1.2* cDNA was cloned into the NotI site of the *vha*-*8p::EGFP* plasmid. The expression vectors *vha*-*8p::ARF*-*1.2(T31N)::EGFP* (dominant negative form) and *vha*-*8p::ARF*-*1.2(Q71L)::EGFP* (constitutively active form) were generated by site-directed mutagenesis with the In-fusion system (Clontech and Takara). The full-length cDNA of *lmp*-*1* and partial cDNA of *aman*-*2* (containing the coding sequence of the first 88 amino acids) were cloned into the NotI site of the *vha*-*8p::EGFP* plasmid to generate *vha*-*8p::LMP*-*1::EGFP* (lysosome/basal membrane) and *vha*-*8p::AMAN*-*2(82aa)::EGFP* (Golgi), respectively. To generate N-terminal fusion constructs, pFX_vha-8p_VenusT(N) was constructed by subcloning the *vha*-*8* promoter into pFX_VenusT(N), and the full-length cDNA of *rab*-*5*, *rab*-*7*, *rab*-*11.1*, and *rme*-*1d* were cloned into the pFX_vha-8p_VenusT(N) to generate *vha*-*8p::VENUS::RAB*-*5* (early endosome), *vha*-*8p::VENUS::RAB*-*7* (late endosome), *vha*-*8p::VENUS::RAB*-*11.1* (recycling endosome), and *vha*-*8p::VENUS::RME*-*1d* (recycling endosome), respectively. To construct *sulp*-*4p::SULP*-*4::EGFP* and *sulp*-*8p::SULP*-*8::EGFP* plasmids, 7.4- and 6.6-kb genomic DNA fragment containing the 5′ upstream promoter and the entire CDS were amplified, and cloned into the pFX_EGFPT plasmid. To generate extrachromosomal (Ex) transgenic animals, these plasmids were injected into N2 at 10 ng/μl with an injection marker *myo*-*2p::dsredm* (at 20 ng/μl) and pBluescript (at 170 ng/μl).

### Bacterial RNAi feeding

RNA interference (RNAi) was carried out by feeding animals dsRNA-producing bacteria, as previously described [33], with some modifications. Briefly, P0 animals at the L4 stage were transferred to plates containing RNAi-bacteria grown on NGM containing 100 µg/ml ampicillin and 1 mM isopropyl-beta-d-thiogalactopyranoside (IPTG). The animals were cultured at 20 °C (or 15 °C in cold tolerance assays) until the F1 animals developed into young adults. F1 animals were used for the subsequent assays so that the knockdown was effective from embryonic stages. For the feeding RNAi screen, post-embryonic RNAi was simultaneously performed. In this case, synchronized animals at L1–L2 stage were transferred to the feeding RNAi plates and cultured until the transferred animals became young adults. For the screening using *tmIs807[hmit*-*1.2p::egfp]* transgenic animals, modified NGM plates that contained ampicillin, IPTG, and fourfold NaCl (200 mM final) were used to induce EGFP expression. The RNAi sub-library (Table S1) for membrane traffic-associated genes was prepared from the Ahringer Library.

### Microscopy and the size measurement of vacuoles

Differential interference contrast (DIC) and fluorescence images were obtained using a BX51 microscope that was equipped with a DP30BW CCD camera (Olympus, Japan). The size measurement of vacuoles was examined by processing fluorescence micrographs with ImageJ (Rasband, W.S., US National Institutes of Health, Bethesda, MD, http://rsb.info.nih.gov/ij/). Investigators were not blinded to the treatment groups performed the image analysis.

### Assays for osmotic stress sensitivity

Synchronized L1 animals (day 1) were cultured on nematode growth medium (NGM) plates containing 51 mM NaCl (normal condition) or 255 mM NaCl (hyperosmotic condition) at 20 °C until they reached 5 days of age (day 5). Body size was determined every day by measuring the projected area of the worm body. The images were taken using a BX51 microscope that was equipped with a DP73 CCD camera (Olympus, Japan) and analyzed using ImageJ software (Rasband, W.S., US National Institutes of Health, Bethesda, MD, http://rsb.info.nih.gov/ij/).

### Transmission electron microscopy

Wild-type or mutant young adults were fixed with 2% paraformaldehyde and 2% glutaraldehyde in 100 mM cacodylate buffer at 4 °C. Transmission electron microscopy was performed by the Hanaichi Ultrastructure Research Institute Co. (Okazaki, Japan). Briefly, fixed samples were postfixed for 2 h with 2% osmium tetroxide in 100 mM cacodylate buffer, followed by dehydration and infiltration with epoxy resin (TAAB, UK). Ultrathin sections of the surface area were analyzed using an electron microscope (H-7600, HITACHI, Japan).

### Electronic supplementary material

Below is the link to the electronic supplementary material.

Supplementary material 1 (PDF 11890 kb)

Supplementary material 2 (PDF 4798 kb)

Supplementary material 3 (DOCX 55 kb)

Supplementary material 4 (XLSX 39 kb)
