# Supplementary figures and images for: The small GTPase ARF-1.2 is a regulator of unicellular tube formation in Caenorhabditis elegans
Source: J Physiol Sci. 2018 Apr 27;69(1):47–56. doi: 10.1007/s12576-018-0617-5 (PMC10717417; doi:10.1007/s12576-018-0617-5)

Wild-type

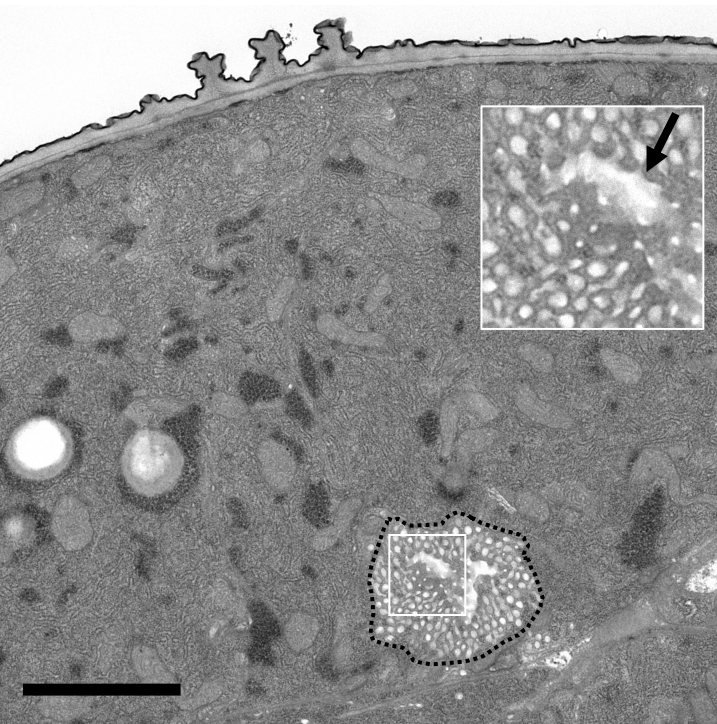

*arf-1.2* (ok796)

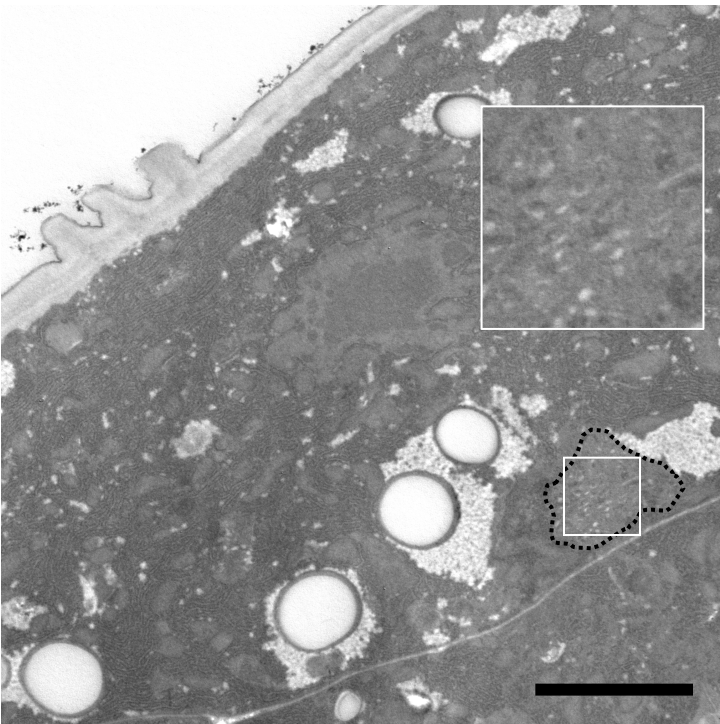

Supplement: Supplementary file 1 — Supplementary material 1 (PDF 11890 kb) [file 12576_2018_617_MOESM1_ESM.pdf]
