# Supplementary material for: The small GTPase ARF-1.2 is a regulator of unicellular tube formation in Caenorhabditis elegans
Source: J Physiol Sci. 2018 Apr 27;69(1):47–56. doi: 10.1007/s12576-018-0617-5 (PMC10717417; doi:10.1007/s12576-018-0617-5)
Supplement: Supplementary file 2 — Supplementary material 2 (PDF 4798 kb) [file 12576_2018_617_MOESM2_ESM.pdf]

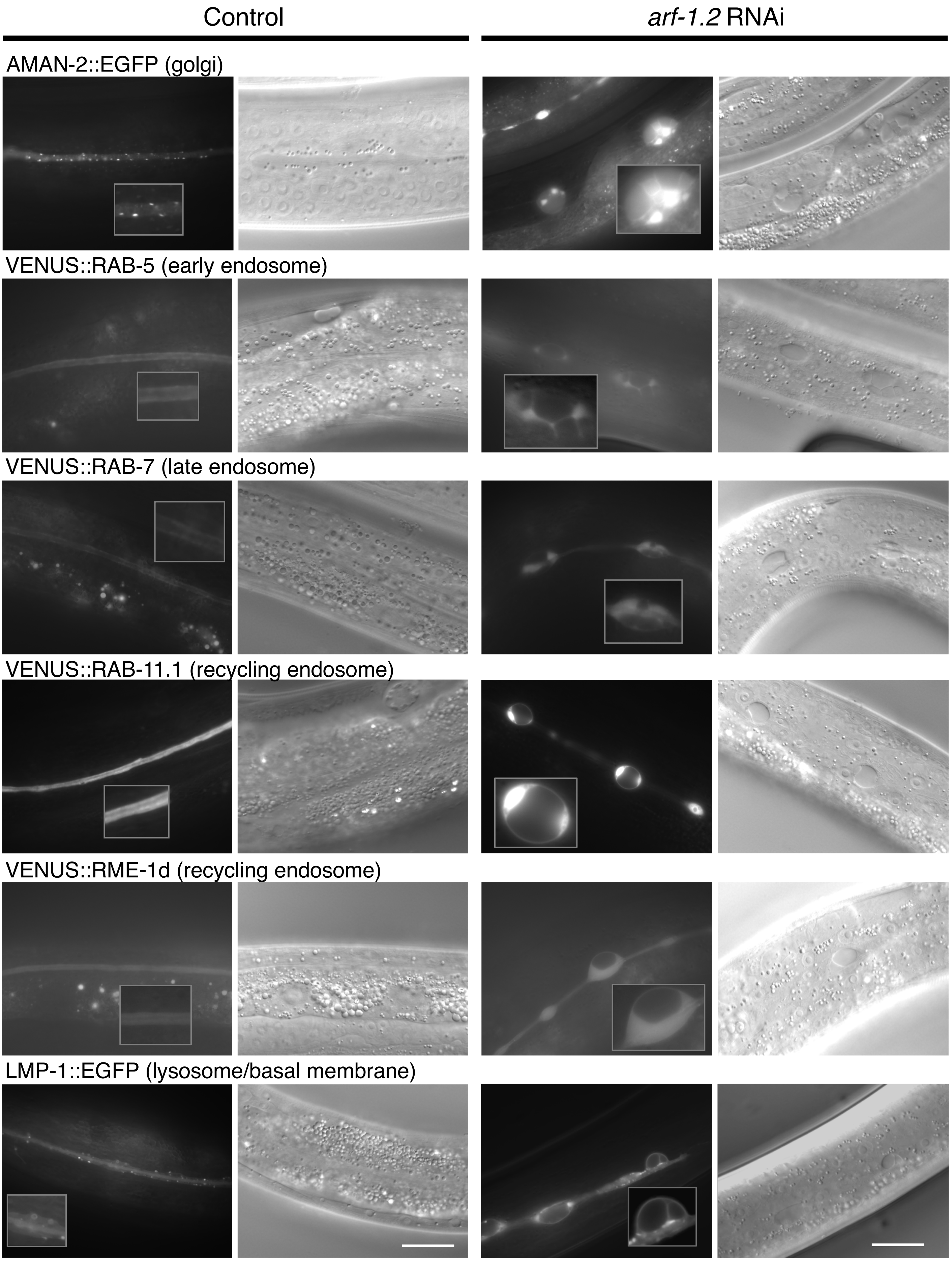

Control
arf-1.2 RNAi
AMAN-2::EGFP (golgi)
VENUS::RAB-5 (early endosome)
VENUS::RAB-7 (late endosome)
VENUS::RAB-11.1 (recycling endosome)
VENUS::RME-1d (recycling endosome)
LMP-1::EGFP (lysosome/basal membrane)
