# Supplementary material for: The small GTPase ARF-1.2 is a regulator of unicellular tube formation in Caenorhabditis elegans
Source: J Physiol Sci. 2018 Apr 27;69(1):47–56. doi: 10.1007/s12576-018-0617-5 (PMC10717417; doi:10.1007/s12576-018-0617-5)
Supplement: Supplementary file 3 — Supplementary material 3 (DOCX 55 kb) [file 12576_2018_617_MOESM3_ESM.docx]

**Supplemental Figure Legends**

**Figure S1** Transmission electron micrograph (TEM) of the *arf-1.2* mutant exhibited poorly defined lumen and canaliculi

TEMs of the wild-type and *arf-1.2* (*ok796*). Black dotted lines indicate the excretory cells. The excretory cells are also shown enlarged in insets. In the wild-type animal, the lumen (arrow) was surrounded by well-structured canaliculi. In the *arf-1.2* animal, the luminal structure was invisible and canaliculi were poorly defined. Scale bars = 2 µm.

**Figure S2** Organellar marker analysis

Transgenic animals that express organellar marker were treated by control and *arf-1.2* RNAi. Fluorescence (left) and DIC (right) images. Enlarged images are shown in insets. Scale bars = 20 µm.
